# Micropipette aspiration as a tool for single-particle X-ray imaging and diffraction

**DOI:** 10.1107/S1600577523003685

**Published:** 2023-05-26

**Authors:** Hendrik Bruns, Hannes Hoeppe, Ewen Bellec, Steven Leake, Markus Osterhoff, Tim Salditt

**Affiliations:** a Georg-August-Universität Göttingen, Institut für Röntgenphysik, Friedrich-Hund-Platz 1, 37077 Göttingen, Germany; b ESRF – The European Synchrotron, X-ray Nanoprobe Group, 71 Avenue des Martyrs, 38000 Grenoble, France; NSRRC, Taiwan

**Keywords:** micropipette aspiration, holographic X-ray imaging, single-particle diffraction, sample delivery of biophysical samples

## Abstract

A sample environment and manipulation tool is presented for single-particle X-ray experiments in an aqueous environment. The system is based on a single water droplet, positioned on a substrate that is structured by a hydrophobic and hydrophilic pattern to stabilize the droplet position. The substrate can support several droplets at a time. Evaporation is prevented by covering the droplet by a thin film of mineral oil. In this windowless fluid which minimizes background signal, single particles can be probed and manipulated by micropipettes, which can easily be inserted and steered in the droplet. Holographic X-ray imaging is shown to be well suited to observe and monitor the pipettes, as well as the droplet surface and the particles. Aspiration and force generation are also enabled based on an application of controlled pressure differences. Experimental challenges are addressed and first results are presented, obtained at two different undulator endstations with nano-focused beams. Finally, the sample environment is discussed in view of future coherent imaging and diffraction experiments with synchrotron radiation and single X-ray free-electron laser pulses.

## Abstract 2

Micropipette aspiration has been combined with a windowless hydrated sample environment for single-particle X-ray analysis. A water droplet serves as a ‘chamber’, covered by a mineral oil to prevent evaporation. In the droplet, particles can be aspirated and manipulated by one or two micropipettes. The system was evaluated on two undulator beamlines, using grains, giant lipid vesicles, colloids and macrophages as test objects. Holographic near-field images were recorded.

## 1. Introduction

The recent advent of single-particle X-ray analysis, with highly brilliant synchrotron and X-ray free-electron laser (XFEL) radiation (Miao *et al.*, 1999 ▸; Gaffney & Chapman, 2007 ▸; van der Schot *et al.*, 2015 ▸; Mancuso *et al.*, 2013 ▸), has created a new need for suitable sample delivery tools. For biophysical experiments, the object has to be positioned in the beam, while remaining in a well defined hydrated environment. Liquid jet and droplet technology (aerosol jets) allow for random injection of particles within an evaporating aerosol droplet. This approach has been demonstrated for viruses (Seibert *et al.*, 2011 ▸; Ekeberg *et al.*, 2015 ▸), as well as colloids, vesicles, bacteria (Seibert *et al.*, 2010 ▸) and even eukaryotic cells (Kimura *et al.*, 2014 ▸; Hantke *et al.*, 2014 ▸). Further – while in flight – the object can be probed only once by a single shot. This is well suited for single-pulse XFEL investigations, implementing the ‘diffract before destroy’ or ‘diffract before damage’ principle, to circumvent radiation damage. The Eulerian angles of the injected particles are randomly distributed, and a sufficiently high number of copies of identical particles have to be probed for three-dimensional (3D) structural analysis. Contrarily, for single non-identical particles, approaches based on sequential imaging at different well controlled rotation angles and positions in the beam may be preferred. This can, for example, be favorably implemented with highly brilliant undulator radiation at synchrotron radiation (SR) sources.

Aside from aerosol or droplet injection, sample delivery for XFEL or synchrotron experiments can also be implemented by channel microfluidics (Denz *et al.*, 2018 ▸; Ghazal *et al.*, 2016 ▸), or micro-liquid enclosure arrays (MLEAs) (Kimura *et al.*, 2014 ▸; Suzuki *et al.*, 2020 ▸). Using microfluidics, the sample can be continuously replenished and the total thickness of the aqueous environment can be kept small, in favor of transmission. However, background signals (scattering or phase variations) of the window materials defining the channels can be challenging in microfluidics (see Hémonnot & Köster, 2017 ▸; Saldanha *et al.*, 2017 ▸), while they do not seem to pose any problems in the MLEA approach.

If, in addition, the particle shall be probed in view of its mechanical response, forces have to be applied on a single particle within the X-ray adapted environment and simultaneously to the X-ray probe.

For this purpose, optical tweezer technology (Ashkin, 1970 ▸, 1978 ▸; Ashkin & Dziedzic, 1987 ▸) was previously implemented for X-ray experiments. Single particles were trapped in an optical tweezer and studied by X-ray diffraction (Cojoc *et al.*, 2007 ▸; Santucci *et al.*, 2011*a* ▸,*b* ▸; Cojoc *et al.*, 2010 ▸), X-ray fluorescence (Vergucht *et al.*, 2015*a* ▸,*b* ▸), as well as by coherent diffraction imaging (Gao *et al.*, 2016 ▸). To avoid significant motion of the particle in the optical potential and to increase the trapping force, counter-propagating laser beams (Guck *et al.*, 2000 ▸) with typical laser powers of 100 mW, up to 1 W, can be used, exerting forces of the order of 100 pN. This so-called optical stretcher prevents a trapped particle from diffusing and can also be used to study the elastic properties of soft objects, such as colloids, biological cells or lipid vesicles (Delabre *et al.*, 2015 ▸; Solmaz *et al.*, 2012 ▸). We have recently explored the experimental capabilities of an optical stretcher as a sample delivery system for X-ray diffraction and coherent imaging, using polymer beads and labeled biological cells (macrophages) as test samples (Nicolas *et al.*, 2018 ▸). From X-ray holograms recorded at different angles of a trapped cell in a microfluidic channel, a 3D reconstruction of the electron density was obtained. Currently, this approach is used to trap giant unilamellar vesicles (GUVs) and to exert forces upon them. One drawback, however, of the microfluidic channels can be the relatively high background scattering signal, depending on the window materials used. In contrast to thinned glass, optically transparent polymer materials used in microfluidics often exhibit substantial background signals in the X-ray regime. For this reason, the optical stretcher has been implemented with glass capillaries and without (index-matching) polymer layers, resulting in optimized signal-to-noise and total transmission. However, the stretcher is not suitable for destructive single-pulse experiments, since the window materials can easily be damaged. Unlike the sample, the windows cannot be easily replenished. Furthermore, optical forces are not suitable for all types of samples and manipulation.

In this work, we combine aspects of both the droplet-based environment and the controlled trapping and force generation. To this end, we replace the microfluidic channel with its plastic or glass windows by a free droplet of well defined size and position. We prevent evaporation by using a thin film of mineral oil to cover the droplet. Trapping and manipulation of a particle within this environment is enabled by one or two micropipettes, which can easily be inserted in the droplet. Unlike optical forces, which would not work in the presence of curved interfaces, forces by the micropipettes can be easily exerted by means of aspiration to trap a particle as well as by simple pushing with a second pipette. We implement such a setup and test it on two different beamlines equipped with nano-focus hard X-ray optics, namely ID01 at the European Synchrotron Radiation Facility (ESRF) in Grenoble, France, and at the GINIX endstation of beamline P10 at the PETRA III storage ring of DESY in Hamburg, Germany. As examples, we show holographic images of the micro-pipettes themselves, as well as aspirated grains, colloidal beads and macrophage cells, after phagocyting (incorporating) barium sulfate particles.

Following this introduction, the experiment is described in Section 2 along with the associated methods, including the micropipette system, droplet mounting and integration into the beamline. Section 3 presents illustrative results obtained for particles, colloids and macrophage cells, before the article closes in Section 4 with a brief conclusion and outlook.

## 2. Experiment

### 2.1. Micropipette aspiration system

**Figure 1:**
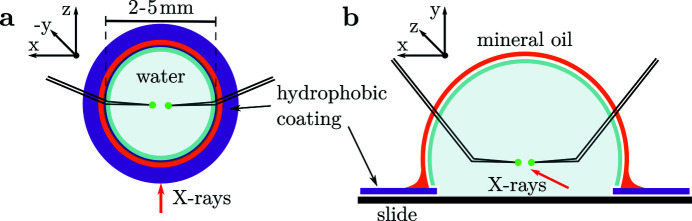
(*a*) Sketch of the micropipette sample environment (top view) on a glass slide. The sample (green) is held by micropipettes with 30° tilted tips, inserted into the sample solution. The droplet is stabilized by a hydrophobic PTFE pattern, or a mini pap pen coating on the supporting glass slide. (*b*) Cross-sectional view of the sample environment with mineral oil engulfing the aqueous solution to prevent evaporation. The X-ray beam traverses both the thin mineral oil film and the water droplet with the sample. No window materials are involved.

Subsequently, we will discuss the components of the micropipette aspiration setup, and the basic principle of the sample environment, as sketched in Fig. 1 ▸.

The setup is composed of pipettes which are immersed into a droplet containing the sample, which in turn is supported by a diagnostic glass slide. The droplet is engulfed by mineral oil by means of wetting from a reservoir close to the droplet.

We use micropipettes pulled from a borosilicate glass capillary of 1 mm diameter with a tip bent to a 30° angle. The tip has either a polished 15 µm opening or a 7 µm inner diameter injection tip (GYNEMED, Germany).

**Figure 2:**
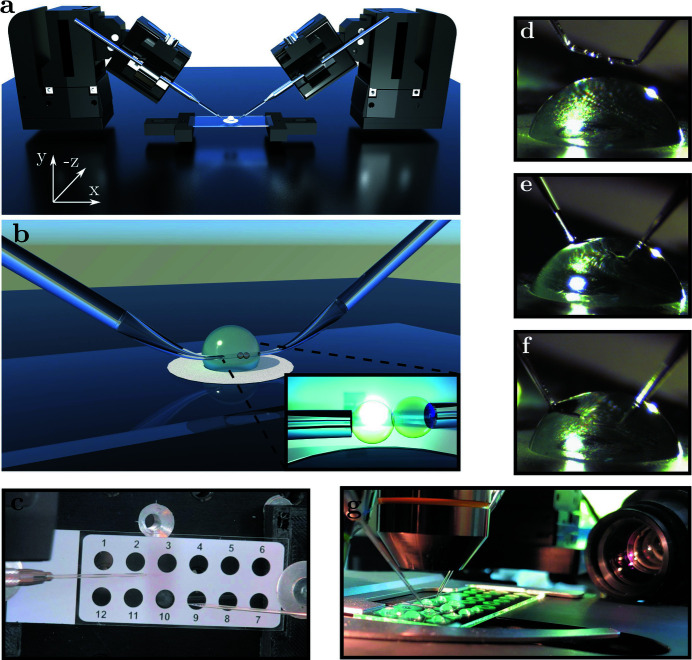
Experimental realization of the micropipette aspiration setup. (*a*) Rendering of the manipulators controlling the movement of the pipettes relative to the sample environment. (*b*) Close-up rendering of pipettes inserted into the sample solution. The inset shows the pipettes bringing two GUVs into contact. (*c*) PTFE-coated diagnostic slide with twelve 5.2 mm-diameter wells, fixing the positions of the sample droplets. (*d*–*f*) Two opposing pipettes (inclined tips with angle of 30°, 80 µm outer diameter, 15 µm inner diameter; GYNEMED, Germany) centered above the droplet and inserted into the droplet. (*g*) The assembly is moved into the focus of an optical microscope with images recorded by an optical network camera.

For support, either a commercially available PTFE-coated slide is used, see Fig. 2 ▸(*c*) (Epredia PTFE Diagnostic Slides with 12 wells 5.2 mm in diameter, Fisher Scientific, Sweden), or a hydrophobic boundary is coated with a pen (Mini PAP PEN, Invitrogen Corporation, USA). Fig. 1 ▸ illustrates how this confines the aqueous sample environment spatially.

Prior to contact with the droplet and the sample, the pipette and the glass slide are passivated by coating with β-casein (cat. C6905, Sigma Aldrich). For this purpose, the pipettes and the slide wells are wetted by a 5 mg ml^−1^ β-casein solution in a 20 m*M*, pH 7.4 TRIS (Cat. 10708976001, Sigma Aldrich, USA) buffer for five minutes (Prévost *et al.*, 2017 ▸). Afterwards, the glassware is rinsed with the buffer solution. For GUVs, a casein coating is particularly important, since they rupture upon contact with the uncoated glass.

### 2.2. Integration into the experimental endstations

**Figure 3:**
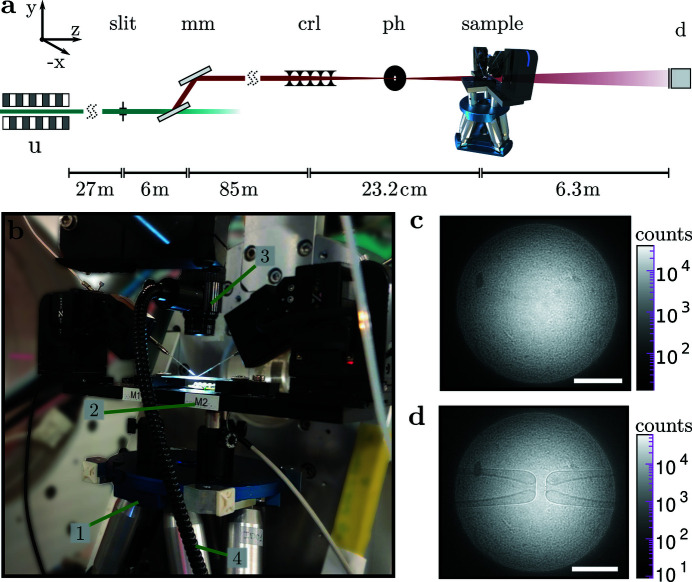
(*a*) Schematic of the ID01 beamline, ESRF, operated at 11 keV. Behind the two U27 undulators (u) and the double-reflection monochromator (mm), the beam is focused by compound refractive lenses (crl). A pinhole (ph) is used as a spatial filter in the focal plane, and a fiber-coupled sCMOS detector (d) captures the images. (*b*) Photograph of the hexapod (1) with the sample stage (2) and an optical microscope (3), supplied by coaxial LED illumination via a fiberglass cable (4). (*c*) Background illumination provided by the setup; (*d*) with pipettes in the field of view. Scale bars: 100 µm.

Next, we describe the experimental implementation, based on the same platform presented in Fig. 2 ▸, which was then installed both at beamline ID01/ESRF, as shown in Figs. 3 ▸ and 4 ▸, as well as at P10/PETRAIII, see Fig. 5 ▸.

In order to aspirate the samples, and bring them into the field of view (FOV) and eventually into contact with each other, they are positioned by remote-controlled piezo motorized micro-manipulators (5 nm resolution, 20 mm range, closed-loop; Sensapex, Finland).

The *x*-axis motor is tilted by 30° to orient the tilted pipette tips parallel to the microscopy slide. A virtual horizontal axis is defined that moves the *x* and *y* motors proportionally to achieve a horizontal movement. This simplifies movement within the FOV and helps to prevent collisions. The controllers communicate with the manipulators via a local network which is accessible from inside the experimental and control hutch.

The aspiration pressure is controlled by pneumatic microinjectors (IM-11-2, NARISHIGE LIFEMED, Japan) transported by plastic tubing to the pipette holder.

Fig. 3 ▸ shows the optical configuration at beamline ID01, ESRF. The 11 keV beam was generated with two U27 undulators. The beam was monochromated with an Si(111) double-reflection monochromator ( 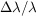 ≃ 10^−4^), and then focused by 50 compound refractive lenses (CRLs) (Be; 50 µm radius at parabola apex; *f* = 186 mm) onto a pinhole (*r* = 5 µm; platinum). The sample environment was positioned on a hexapod stage (BORA, Symétrie, France). Images were recorded with an sCMOS detector (Zyla 5.5, 5M, 6.5 µm pixel size; Andor, UK), fiber coupled to a 15 µm Gadox scintillator. A photograph of the micropipette setup on the sample stage is shown in Fig. 3 ▸(*b*). As can be seen in Figs. 3 ▸(*c*) and 3(*d*), the flat field of the CRL is characterized by a well defined divergence angle (0.052 mrad) with a rather sharp radius defined by the round aperture in front of the CRL. The Be lenses impart a structured phase profile onto the beam, visible as a grainy pattern in the detector plane, see Figs. 3 ▸(*c*). The pinhole inserted in the focal plane of the CRL serves to filter out some of these phase distortions. Despite the remaining wavefront artifacts and intensity variations, the two opposing micro­pipettes are well resolved and contrasted.

**Figure 4:**
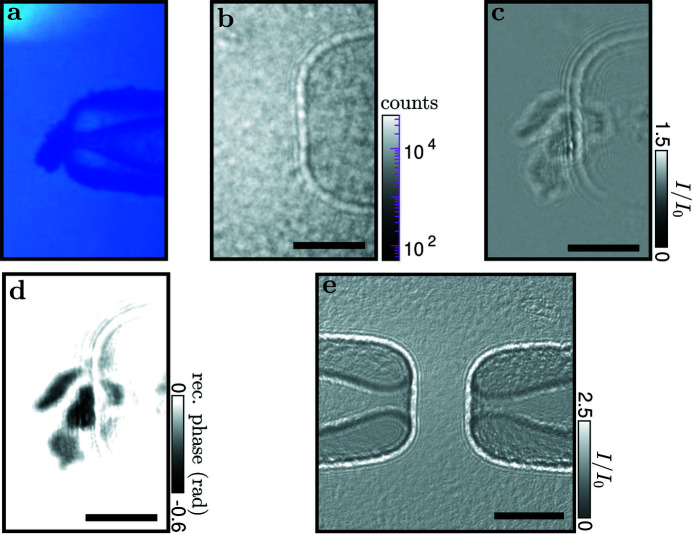
Holographic imaging at ID01. (*a*–*d*) Coffee grain aspirated by a pipette and used as a test object. (*a*) Optical microscope top view showing the sample reaching into the opening. (*b*) Hologram of the aspirated sample. (*c*) Hologram corrected by a flat field containing the pipette. (*d*) Nonlinear Fresnel Tikhonov phase reconstruction (Huhn *et al.*, 2022 ▸; Lohse *et al.*, 2020 ▸) (β/δ = 0.0286, lim1 = 10^−4^, lim2 = 3 × 10^−1^). (*e*) Hologram of GUV imaging attempt not yet showing sufficient contrast. Scale bars: 30 µm.

Fig. 4 ▸ shows that the near-field X-ray images can be used very well to align the system, with Fig. 4 ▸(*a*) showing the optical long-distance microscope pointed at one of the pipettes from above and serving as a reference, such that the optical [Fig. 4 ▸(*a*)] and the X-ray [Fig. 4 ▸(*b*)] field of view coincide. Note that the optical microscope was equipped with a network camera that was accessible in the experimental and control hutches, so that the pipette could be steered by remote control with visual feedback from the online X-ray image and simultaneously the optical camera. As Fig. 4 ▸ also shows, a fairly ‘clean’ holographic image is obtained by division of the flat (empty) which still includes the pipettes; see Fig. 4 ▸(*c*) for an example of an aspirated coffee grain. By phase retrieval, the projected phase of the object is reconstructed, see Fig. 4 ▸(*d*). This is possible for an object with a rather large phase shift like the grain. An attempt to also visualize an aspirated GUV, however, failed, as shown in Fig. 4 ▸(*e*); this is either due to dominating background noise or the residual structures and fine fringes due to the optically strong pipettes screening the weak signal of the lipid bilayer. This is despite the large magnification and the principle visibility of lipids and surfactant interfaces in holographic projection images (Beerlink *et al.*, 2009 ▸, 2012 ▸). Note that the projected phase of the GUV could potentially yield sufficient signal if the flat adhesion zone of two adhering bilayers was perfectly aligned with the optical axis, increasing the projected scattering length difference. For a lipid bilayer, a sufficient phase signal is only accumulated when the normal vector of such an adhesion zone is almost orthogonal to the beam. Future extension of this work could reach this. More progress can be achieved by facilitating alignment, in particular by improving the optical microscope and by better alignment of the rotation center with respect to the X-ray optical axis.

**Figure 5:**
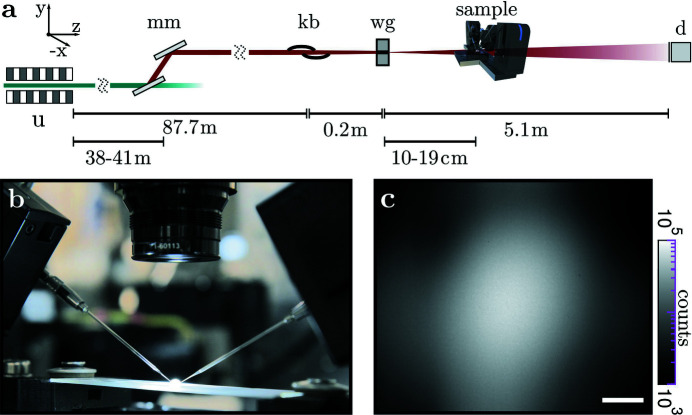
(*a*) Setup schematic of the GINIX endstation at beamline P10, DESY, showing the 5 m U32 undulator (u), the Si(111) double-crystal monochromator (mm), the KB mirrors and the waveguide (wg). The sample is positioned in defocus of the waveguide illumination and magnified inline holograms are recorded by a fiber-coupled sCMOS detector positioned 5.1 m behind the sample. (*b*) Photograph of the sample environment. (*c*) Waveguide empty beam illumination. Scale bar: 50 µm.

Fig. 5 ▸ depicts the configuration at the GINIX endstation at beamline P10, DESY, dedicated to holo-tomography and equipped with a compound Kirkpatrick–Baez (KB) mirror focusing optic and additional X-ray waveguides (Salditt *et al.*, 2015 ▸). The parameters were as follows. The 5 m U32 undulator was operated with the third harmonic at 13.8 keV. The beam was monochromated to 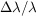 ≃ 10^−4^ by a Si(111) monochromator. The focusing optics consisted of KB mirrors (spot size ∼300 nm × 300 nm). An X-ray waveguide (wg; crossed MoCMo 58.9 nm) was positioned in the focal plane of the KB for further beam confinement resulting in a spot size ≤30 nm (Krüger *et al.*, 2012 ▸). Images were recorded by a sCMOS detector (Zyla 5.5, 5M, fiber coupled to a 15 µm Gadox scintillator, 6.5 µm pixel size; Andor, UK).

## 3. Results

### 3.1. Polystyrene beads

**Figure 6:**
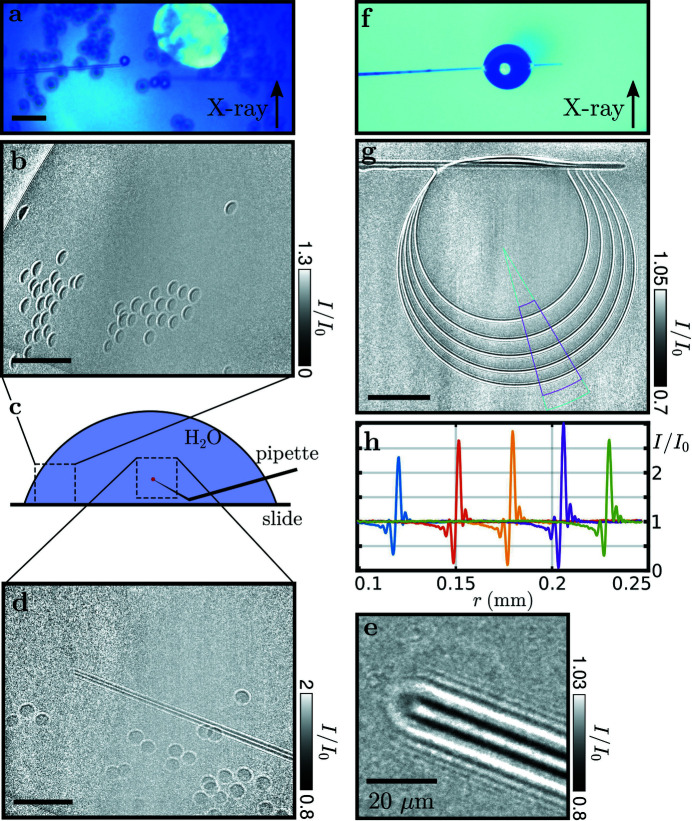
(*a*) Birds-eye-view microscopy image of aspirated polystyrene beads (30 µm diameter). (*b*) Hologram of polystyrene beads sticking out of the water droplet. (*c*) Positions of the images (*b*, *d*) with respect to the droplet. (*d*) Hologram of the pipette with an aspirated bead; several beads accumulate at the air–water interface of the droplet. (*e*) Hologram of the pipette with a bead aspirated at the tip. (*f*) Water droplet suspended from a pipette (top view microscope). (*g*) Six successive holograms (added), showing the evaporation process. (*h*) Water–air interface profiles of the holograms, taken along the radial cross section of the magenta region in (*g*). Scale bars: 100 µm.

Fig. 6 ▸ shows the imaging of *d* = 30 µm polystyrene beads suspended in water as well as the ability to bring liquid droplets of small volume into the X-ray beam. In the optical regime [Fig. 6 ▸(*a*)] the aspirated beads show a high contrast. In the X-ray regime polysterene has a low contrast to water due to a similar electron density of 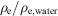 = 1.027 (Schoenfeld *et al.*, 2015 ▸). This means that only the parts extending out of the water are visible in the hologram [Figs. 6 ▸(*b*) and 6(*d*)] with a 1 s illumination time. On the sides of the sample environment [Fig. 6 ▸(*b*)] spherical extensions of the beads are projected to appear as circular segments in the image plane. In the center [Fig. 6 ▸(*d*)] of the droplet these extensions are projected to full circles. A total illumination time of 48 s offers enough contrast to resolve the aspirated bead on the tip of the pipette [Fig. 6 ▸(*e*)].

To suggest another avenue of microfluidic experiments with this setup we situated a small droplet under the pipette [Figs. 6 ▸(*f*)–6(*h*)]. The quick evaporation of such a small water droplet can be compensated by a constant flow of liquid from the pipette. The evaporation process is depicted in Fig. 6 ▸(*g*) where five holograms are added to show five time steps in one image. The water–air interface is averaged along the angle in the magenta slice to form the intensity edge profiles depicted in Fig. 6 ▸(*h*).

### 3.2. Macrophages loaded with barium sulfate

**Figure 7:**
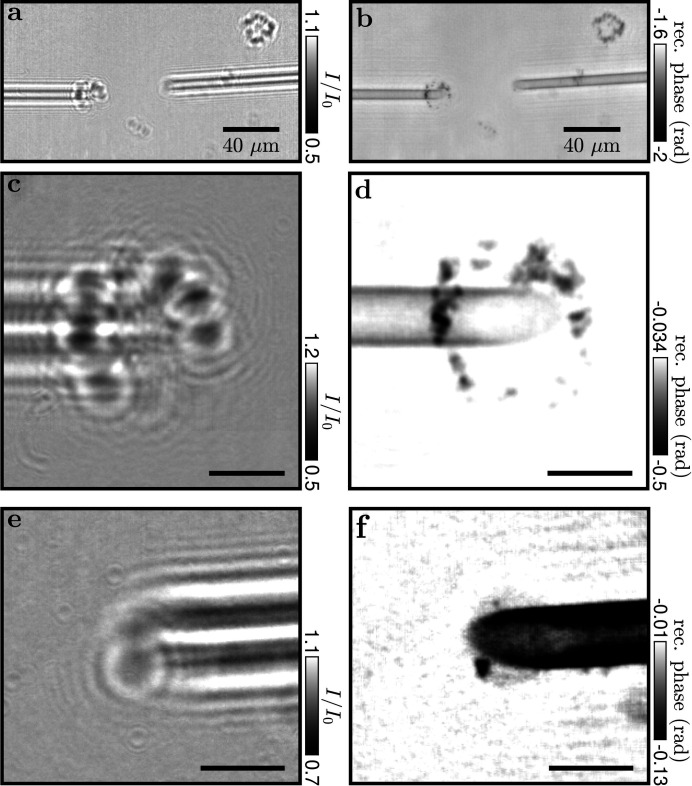
Holograms of aspirated macrophages, stained with barium (BaSO_4_). (*a*) Hologram of opposing pipettes, each having a macrophage aspirated at a wg-to-sample distance of *z*_01_ = 19 cm. (*b*) Tikhonov reconstruction (Huhn *et al.*, 2022 ▸; Lohse *et al.*, 2020 ▸). (*c*) Hologram of the left macrophage. (*d*) Tikhonov reconstruction with a wg-to-sample distance of *z*_01_ = 10 cm. (*e*, *f*) Hologram of the right macrophage. (*f*) Tikhonov reconstruction with a wg-to-sample distance of *z*_01_ = 10 cm. Scale bars: 10 µm.

In order to explore the ability to image single cells with the micropipette setup we use fixed alveolar macrophages from mice labeled with BaSO_4_. In Fig. 7 ▸(*a*) two aspirated and three free-floating macrophages are shown. They are recorded with a focus-to-sample distance of *z*_01_ = 190 mm with a summed illumination time of 40 s. In this case, the droplet was rather large with a diameter of 7 mm, resulting in low transmission of about *T* = 0.26. The hologram is reconstructed using a Tikhonov reconstruction (Huhn *et al.*, 2022 ▸; Lohse *et al.*, 2020 ▸) with the parameters 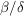 = 0.037, lim1 = 9 × 10^−3^, lim2 = 10^−1^ in Fig. 7 ▸(*b*). We see that the BaSO_4_ particles of the aspirated macrophages are well positioned and thus less smeared out compared with the free-floating cell markers. The same samples are also imaged with a smaller focus-to-sample distance of *z*_01_ = 100 mm for a higher magnification. They are displayed in two zoom-ins on the left pipette with a 54 s exposure time in Fig. 7 ▸(*c*) and the right pipette with a 56 s exposure time in Fig. 7 ▸(*e*). The Tikhonov reconstruction of the left tip [Fig. 7 ▸(*d*)] makes the BaSO_4_ particles differentiable from the pipette tip. The markers are resolved with a Michelson contrast of *c*_m_ = 0.137. The Tikhonov reconstruction ( 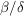 = 0.037, lim1 = 1 × 10^−5^, lim2 = 5 × 10^−3^), see Fig. 7 ▸(*f*), of the right tip shows the outline of the aspirated cell encompassing two visible BaSO_4_ particles.

## 4. Summary, discussion and outlook

In summary, we have presented a ‘windowless’ X-ray compatible sample environment for biophysical experiments in fully hydrated sample environments, suitable both for synchrotron and XFEL experiments. Along with the simple, yet powerful, concept of a liquid droplet capped by mineral oil to avoid evaporation, and positional stabilization by a hydrophobically patterned glass slide, the chamber is equipped with two micropipettes for aspiration and manipulation of one or two objects. The pipettes can be easily inserted into the droplet and positioned by micro-manipulators, monitored by optical and X-ray imaging. The setup was characterized at two different beamlines with different optics for nano-focusing and holographic imaging. The test objects included an aspirated macrophage cell (after BaSO_4_ internalization), aspirated by the pipette, and imaged holographically with subsequent phase retrieval.

**Figure 8:**
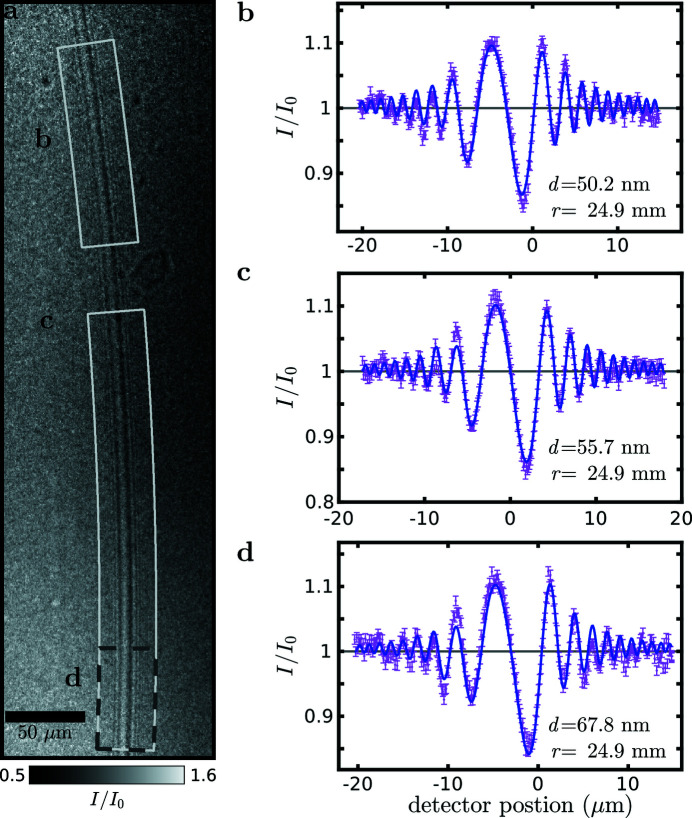
(*a*) Holographic propagation imaging of a so-called black lipid membrane (BLM), *i.e.* lipid membranes spanned over an aperture between two aqueous compartments in a Teflon chamber with polyimide windows for X-ray transmission. Since the lipids system is prepared by ‘painting’ a solution of lipids in decane, the bilayer is still swollen with decane. The exact thickness of the BLM can be determined by analysis of the Fresnel finges. Following Beerlink *et al.* (2009 ▸), we can model the density profile (or equivalently phase profile) and use Fresnel propagation for least-square fitting of the radial intensity profiles (*b*, *c*, *d*). The experimental profiles are shown as a function of radial distance *r* from the center of a circle describing the averaging over a range of azimuthal angles, as indicated in (*a*), to increase the signal-to-noise ratio. Here, the BLM thickness *d* was ranged between 50 nm and 70 nm, but this depends on the configuration, and in particular on the solvent outflow (BLM thinning).

The fact that the micropipettes can be equipped with a pressure gauge and that deformations of a soft object such as a GUV could be observed in the beam has not yet been exploited in this work. This was also due to the fact that visualization of the GUV was not yet successful. The challenge here is to achieve an illumination wavefront as clean as possible, for example by wavefront filtering, as also shown here at the second beamline (P10/PETRAIII), and at the same time to implement suitable alignment procedures and sufficient angular range to bring two adhering lipid bilayers into perfect tangential orientation with respect to the X-ray optical axis. In fact, one specific long-term scientific goal, for which we develop this experimental technology, is to study membrane adhesion (Komorowski *et al.*, 2018 ▸) and the onset of membrane fusion. Note that a system of a single bilayer or two adhering bilayers results in a characteristic magnified holographic fringe pattern which contains information on the nanoscale structure of the system, in particular its density profile (see the sketch in Fig. 8 ▸ [App appa]).

At the same time we anticipate that other researchers may find the system presented of interest for entirely different experiments. A line of droplets, prevented from evaporation by the mineral oil capping, could bring particles or analytes to an intense X-ray beam. Transmission can be increased and background signals can be suppressed by optimizing the droplet sizes. Contrarily, window materials of microfluidic channels cannot be scaled accordingly, and may pose a stronger limit regarding phase and background variations.

Micropipettes, which can be inserted into droplets but not into microfluidic channels, are ideal for aspiration to tightly position a particle, but they can also simply be used to fill the droplets (with small particles or analytes) before X-ray measurements. In some experiments, the pressure difference between chamber and micropipette may serve as a control parameter, for example to study the formation of an aspirated object, or to induce flow. With a remote-controlled pipette pressure the size of the droplet can also be adjusted. While we used rather large droplets of several millimetres in diameter in this proof of concept, the droplet diameter could be scaled over a wide range, even down to sub-micrometre scales (Sheyfer *et al.*, 2023 ▸), and therefore could also be used as a sample environment without micropipette aspiration.

## Appendix A Imaging of a bulged lipid bilayer spanned over an aperture

Fig. 8 ▸ shows Fresnel propagation images of black lipids membranes that were spanned over an aperture, with the beam propagating tangentially to the bulged membrane, following Beerlink *et al.* (2009 ▸). From the analysis of the fringes, the normal phase profile can be obtained. These images were acquired in the same beam time as the micropipette results above, and confirm the feasibility of imaging lipid membrane, as soon as the propagation distance within the bilayer creates sufficient contrast.
